# *Cis*-1,4-Polymerization of Isoprene by 1,3-Bis(oxazolinymethylidene)isoindoline-Ligated Rare-Earth Metal Dialkyl Complexes

**DOI:** 10.3390/polym9100531

**Published:** 2017-10-20

**Authors:** Chao Yu, Dahai Zhou, Xiangqian Yan, Fei Gao, Li Zhang, Shaowen Zhang, Xiaofang Li

**Affiliations:** Key Laboratory of Cluster Science of Ministry of Education, School of Chemistry and Chemical Engineering, Beijing Institute of Technology, 5 South Zhongguancun Street, Haidian District, Beijing 10081, China; strongfire520@126.com (C.Y.); zdh20142801@163.com (D.Z.); hsdyxq@126.com (X.Y.); gaofeiring@163.com (F.G.); lizhang751647020@163.com (L.Z.)

**Keywords:** rare-earth metal complexes, 1,3-bis(oxazolinymethylidene)isoindoline ligand, polymerization, isoprene, *cis*-1,4 selectivity

## Abstract

A series of novel chiral nonmetallocene pincer-type rare-earth metal dialkyl complexes bearing the chiral monoanionic tridentate *C*_2_-symmetric 1,3-bis(oxazolinymethylidene)isoindoline (BOXMI-H) ligand (BOXMI)Ln(CH_2_SiMe_3_)_2_ 1–3 (1: Ln = Sc, yield = 57%; 2: Ln = Lu, yield = 55%; 3: Ln = Y, yield = 62%) have been prepared in moderate yields via the acid-base reaction between the BOXMI ligand and rare-earth metal tri(trimethylsilylmethyl) complexes. The X-ray diffractions show that both of the complexes 1 and 2 contain one BOXMI ligand and two trimethylsilylmethyl ligands, adopting a distorted trigonal bipyramidal configuration. In the presence of a cocatalyst such as borate and AlR_3_, these complexes 1–3 exhibit high activities of up to 6.8 × 10^4^ (g of polymer)/(mol_Ln_ h) and high *cis*-1,4 selectivities of up to 97% in the polymerization of isoprene in toluene, yielding the *cis*-1,4-polyisoprenes with heavy molecular weights (*M*_n_ of up to 710,000 g/mol) and bimodal molecular weight distributions (*M*_w_/*M*_n_ = 2.0–4.5).

## 1. Introduction

**Chart 1:**
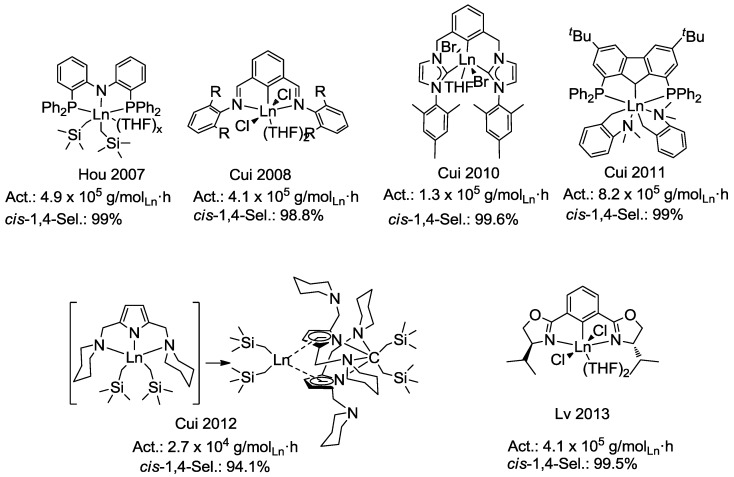
The previous pincer-type rare-earth metal catalysts bearing tridentate *C*_2_-symmetric chelating ligand.

The development of highly efficient and selective organometallic catalysts is a permanent topic for olefin polymerization, which brings new opportunities for the synthesis of high-performance (co)polymers with precisely controlled structures and excellent properties [1,2,3,4,5,6]. Recently, *cis*-1,4-polyisoprene (*C*PIP) has attracted much attention from the academia and industry in the view that it is the principal component of natural rubber and serves as a strategically important elastomer used for pneumatic tires [7,8,9,10,11]. In general, the practical and most common synthesis method of *C*PIP is the coordination–insertion polymerization of isoprene (IP) by using homogeneous organometallic catalysts based on rare-earth metals [12,13,14,15,16,17,18]. So far, a large number of the rare-earth metal catalyst precursors bearing a different chelating ligand have been reported for the *cis*-1,4-polymerization of IP [19,20,21,22,23,24,25,26,27]. Among these, the nonmetallocene pincer-type rare-earth metal complexes bearing the monoanionic tridentate *C*_2_-symmetric ligands usually exhibit both high activities and high *cis*-1,4 selectivities in the polymerization of IP [28,29,30] (Chart 1). In 2007, the rare-earth metal alkyl complexes bearing a bis(phosphinophenyl)amido (PNP) ligand were reported by Hou et al., showing high activities of up to 4.9 × 10^5^ (g of polymer)/(mol_Ln_ h) and high *cis*-1,4 selectivities of up to 99% in the living *cis*-1,4-polymerization of isoprene [31]. In 2008, the aryldiimine (NCN)-ligated rare-earth metal dichlorides were developed by Cui and co-workers. These complexes can serve as catalyst precursors in the *cis*-1,4-polymerization of isoprene with high activities of up to 4.1 × 10^5^ (g of polymer)/(mol_Ln_ h) and high *cis*-1,4 selectivities of around 98.8% [32]. Subsequently, Cui also reported the *cis*-1,4-polymerization of isoprene by using the bis(carbene)phenyl (CCC) rare-earth metal dibromides (activities of up to 1.3 × 10^5^ (g of polymer)/(mol_Ln_ h) and *cis*-1,4 selectivities of up to 99.6%) [33], the bis(phosphino)carbazolide (PNP)-chelated rare-earth metal complexes (activities of up to 8.2 × 10^5^ (g of polymer)/(mol_Ln_ h) and *cis*-1,4 selectivities of up to 99%) [34], and the bis(pyrrolidin-1-yl)pyrrolyl or bis(piperidino)methylene) pyrrolyl [NNN]-ligated rare-earth metal complexes (activities ca. 2.7 × 10^4^ (g of polymer)/(mol_Ln_ h) and *cis*-1,4 selectivities ca. 94.1%) [35]. In 2013, Lu and coworkers described the *cis*-1,4-polymerization of isoprene by use of the bis(oxazolinyl)phenyl (NCN)-ligated rare-earth metal dichlorides with activities of up to 4.1 × 10^5^ (g of polymer)/(mol_Ln_ h) and *cis*-1,4 selectivities of up to 99.5% [36]. By contrast, the rare-earth metal complexes bearing the pincer-type NNN ligand exhibited unsatisfactory activity (<10^5^ (g of polymer)/(mol_Ln_ h)) and *cis*-1,4 selectivity (<94%) in the polymerization of IP [37]. Therefore, it is of great interest to develop higher-efficiency and selective NNN-chelated rare-earth metal complexes for the *cis*-1,4-polymerization of IP.

Recently, we have paid attention to the synthesis of the NNN-ligated rare-earth metal dialkyl complexes and their applications in IP polymerization. In 2013, we reported the synthesis of a series of chiral (S,S)-bis(oxazolinylphenyl)amine ((S,S)-BOPA)-ligated rare-earth metal dialkyl complexes [(S,S)-BOPA]Ln(CH_2_SiMe_3_)_2_ (1–2; 1: Ln = Sc; 2: Ln = Lu). In the presence of an activator with or without a small amount of Al*^i^*Bu_3_, the dialkyl complexes 1 and 2 exhibited very high activities of up to 6.8 × 10^5^ (g of polymer)/(mol_Ln_ h) and *trans*-1,4 selectivities of up to 100% in the quasi-living polymerization of isoprene, yielding *trans*-1,4-PIPs with moderate molecular weights (*M*_n_ = (0.2–1.0) × 10^5^ g/mol) and narrow molecular weight distributions (*M*_w_/*M*_n_ = 1.02–2.66) [38]. Recently, we also reported that the 1,3-bis(2-pyridylimino)isoindoline-ligated rare-earth metal dialkyl complexes showed very high activities of up to 1.9 × 10^6^ (g of polymer)/(mol_Ln_ h) and high *cis*-1,4 selectivities of >99% in the polymerization of isoprene in the presence of an activator and AlR_3_, affording *C*PIP with heavy molecular weights (*M*_n_ up to 610,000 g/mol) and narrow to moderate molecular weight distributions (*M*_w_/*M*_n_ = 1.26–2.08) [39]. These results demonstrated that the effective adjustment of the skeleton of the pincer-type NNN ligand have an important impact on the catalyst performance of these rare-earth metal complexes in IP polymerization, which rouses our interests to explore more NNN-ligated rare-earth metal dialkyl complexes and to detect their catalytic performance in the selective polymerization of IP. 1,3-Bis(oxazolinymethylidene)isoindoline (BOXMI-H) ligand, which has structural characteristics of both the (S,S)-bis(oxazolinylphenyl)amine and 1,3-bis(2-pyridylimino)isoindoline ligands, is an interesting chiral tridentate *C*_2_-symmetric NNN ligand for the organometallic complex based on transition metals for the asymmetric reaction [40,41,42,43]. Until now, the BOXMI-H-ligated rare-earth metal complexes have never been reported, and their applications for the coordination–insertion polymerization of olefin have never been investigated, as far as we are aware. Herein, we report the synthesis of three pincer-type BOXMI-H-ligated rare-earth metal dialkyl complexes (BOXMI)Ln(CH_2_SiMe_3_)_2_ 1–3 (1: Ln = Sc; 2: Ln = Lu; 3: Ln = Y) via the acid–base reaction between the BOXMI-H ligand and rare-earth metal trialkyl complexes. These complexes 1–3 exhibited high activities of up to 6.8 × 10^4^ (g of polymer)/(mol_Ln_ h) and high *cis*-1,4 selectivities of up to 97% in the IP polymerization in toluene, affording *cis*-1,4-polyisoprenes with heavy molecular weights (*M*_n_ of up to 710,000 g/mol) and bimodal molecular weight distributions (*M*_w_/*M*_n_ = 2.0–4.5).

## 2. Experiment

### 2.1. Materials and Method

All catalysts and the polymerization procedure were carried out in a nitrogen-filled MBraun glovebox. [Ph_3_C][B(C_6_F_5_)_4_], [PhMe_2_NH][B(C_6_F_5_)_4_], and B(C_6_F_5_)_3_ were purchased from J&K Chemical (Beijing, China). LiCH_2_SiMe_3_ (1.0 M solution in pentane) and LnCl_3_ were purchased from Aldrich (St. Louis, MO, USA). Al*^i^*Bu_3_ (1.1 M solution in hexane), AlMe_3_ (1.0 M solution in Toluene), AlEt_3_ (0.6 M solution in heptane), Phthalimides, (Carbethoxymethylene)triphenylphosphorane, (*S*)-amino alcohol, NaH, PPh_3_, Et_3_N, CCl_4_, Na_2_SO_4_, CaH_2_, dichloromethane, petroleum ether, and methanol were obtained from Energy Chemistry (Shanghai, China). BOXMI-H ligand [40] and Ln(CH_2_SiMe_3_)_3_(THF)_2_ [44] were prepared according to the literature. Isoprene was purchased from J&K Chemical (Beijing, China), and dried through CaH_2_. Toluene, THF, and hexane were purified by a solvent purification system (SPS-800, Mbraun, Shanghai, China) and dried over Na in the glovebox. The deuterated solvents C_6_D_6_ (99.6 atom% D) and CDCl_3_ (99.8 atom% D) were purchased from Cambridge Isotope.

Elemental analyses, ^1^H NMR and ^13^C NMR, of rare-earth metal complexes were performed according to the literature [38]. Polyisoprene samples of gel permeation chromatography (GPC) and differential scanning calorimetry (DSC) measurements were conducted according to the literature [38].

The test method of crystals of complexes 1 and 2 was performed according to the literature [38].

Crystallographic data of complexes 1 and 2 (excluding structure factors) have been deposited to the Cambridge Crystallographic Data Centre as supplementary publication nos. CCDC-1539945 (1) and 1536645 (2), containing the supplementary crystallographic data for this paper. These data can be obtained free of charge via https://www.ccdc.cam.ac.uk/structures/ from the Cambridge Crystallographic Data Centre.

### 2.2. Synthesis of Chiral BOXMI-H-Ligated Rare-Earth Metal Dialkyl Complexes

Here we outline the synthesis of the BOXMI-H-ligated rare-earth metal dialkyl complex (BOXMI)Sc(CH_2_SiMe_3_)_2_ (**1**). To a colorless toluene solution (8.0 mL) of Sc(CH_2_SiMe_3_)_3_(THF)_2_ (208 mg, 0.461 mmol) was added a solution of the BOXMI-H ligand (200 mg, 0.461 mmol) in toluene (10.0 mL) at room temperature. The reaction mixture was stirred at room temperature for 4 h. After removal of the solution in vacuo, the resulting solid was recrystallized from hexane/toluene at −30 °C to give **1** (171 mg, yield ca. 57%). ^1^H NMR (400 MHz, C_6_D_6_) δ: 7.25–7.23 (m, 6H), 7.18–7.14 (m, 4H), 7.11–7.09 (m, 2H), 5.79 (s, 2H), 5.56 (dd, 2H, *J* = 4 Hz, 8 Hz), 4.27 (t, 2H, *J* = 8 Hz), 3.91 (dd, 2H, *J* = 4 Hz, 8 Hz), 0.17 (d, 2H, *J* = 12 Hz), 0.02 (s, 18H, CH_2_Si(C*H*_3_)_3_), and −0.54 (d, 2H, *J* = 12 Hz,). ^13^C NMR (100 MHz, C_6_D_6_) δ: 173.71, 162.61, 142.62, 138.31, 130.10, 129.41, 128.63, 127.93, 121.10, 82.76, 75.81, 69.71 and 3.46. Anal. Calcd (%) for C_36_H_44_N_3_O_2_ScSi_2_: C, 60.33; H, 6.80; N, 6.45. Found: C, 60.31; H, 6.78; N, 6.46.

The following outlines the synthesis of BOXMI-H-ligated rare-earth metal dialkyl complex (BOXMI)Lu(CH_2_SiMe_3_)_2_ (**2**). To a colorless toluene solution (5.0 mL) of Lu(CH_2_SiMe_3_)_3_(THF)_2_ (268 mg, 0.461 mmol) was added a solution of the BOXMI-H ligand (200 mg, 0.461 mmol) in toluene (10.0 mL) at room temperature. The reaction mixture was stirred at room temperature for 4 h. After removal of the solution in vacuo, the resulting solid was recrystallized from hexane/toluene at −30 °C to give **2** (198 mg, yield ca. 55%). ^1^H NMR (400 MHz, C_6_D_6_) δ: 7.17–7.12 (m, 10H), 7.09–7.07 (m, 2H), 6.92–6.90 (m, 2H), 5.72 (s, 2H), 5.41 (dd, 2H, *J* = 4 Hz, 8 Hz), 4.19 (t, 2H, *J* = 8 Hz), 3.84 (dd, 2H, *J* = 4 Hz, 8 Hz), 0.05 (s, 18H), −0.62 (d, 2H, *J* = 12 Hz), and −1.44 (d, 2H, *J* = 12 Hz). ^13^C NMR (100 MHz, C_6_D_6_) δ: 170.81, 163.41, 141.92, 138.85, 130.06, 129.48, 128.81, 127.97, 121.08, 82.50, 75.65, 68.91, 43.56, and 4.00. Anal. Calcd (%) for C_36_H_44_N_3_O_2_LuSi_2_: C, 55.30; H, 5.67; N, 5.37. Found: C, 55.32; H, 5.71; N, 5.41.

The following outlines the synthesis of BOXMI-H-ligated rare-earth metal dialkyl complex (BOXMI)Y(CH_2_SiMe_3_)_2_ (**3**). To a colorless toluene solution (5.0 mL) of Y(CH_2_SiMe_3_)_3_(THF)_2_ (228 mg, 0.461 mmol) was added a solution of the BOXMI-H ligand (200 mg, 0.461 mmol) in toluene (10.0 mL) at room temperature. The reaction mixture was stirred at room temperature for 4 h. After removal of the solution in vacuo, the resulting solid was recrystallized from hexane/toluene at −30 °C to give **3** (199 mg, yield ca. 62%). ^1^H NMR (400 MHz, C_6_D_6_) δ: 7.18–7.12 (m, 12H), 7.09–7.07 (m, 2H), 6.92–6.89 (m, 2H), 5.72 (s, 2H), 5.40 (dd, 2H, *J* = 4 Hz, 8 Hz), 4.17 (t, 2H, *J* = 8 Hz), 3.81 (dd, 2H, *J* = 4 Hz, 8 Hz), 0.05 (s, 18H), −0.41 (d, 2H, *J* = 12 Hz), and −1.30 (d, 2H, *J* = 12 Hz). ^13^C NMR (100 MHz, C_6_D_6_) δ: 170.80, 163.13, 141.73, 138.9, 129.97, 129.56, 127.97, 121.00, 82.26, 75.35, 68.85, 37.29, 37.08, and 3.88. Anal. Calcd (%) for C_36_H_44_N_3_O_2_YSi_2_: C, 62.14; H, 6.37; N, 6.04. Found: C, 62.10; H, 6.35; N, 6.01.

### 2.3. A Typical Procedure for Isoprene (IP) Polymerization

A detailed polymerization procedure of isoprene is described here as a typical example. In a glovebox at 25 °C, to a toluene solution (8 mL) of Al*^i^*Bu_3_ (181 μL, 1.1 M, 200 μmol) was added a toluene solution (8 mL) of complex 1 (0.013 g, 20 μmol), a toluene solution (2.5 mL) of [Ph_3_C][B(C_6_F_5_)_4_] (0.018 g, 20 μmol), and isoprene (0.27 g, 4 mmol) in succession. In a 50 mL round bottom flask, the reaction mixture then became viscous rapidly. The flask was taken outside after 0.5 h, and then the solution was added to ethanol (50 mL, containing 5% butylhydroxytoluene (BHT) as a stabilizing agent) to quench the reaction mixture. The obtained polymer was washed three times by ethanol and dried under vacuum at 40 °C to a constant weight (0.23 g, yield ca. 85%). The resulting polymer was soluble in THF and chloroform at room temperature. The isomer contents of the polyisoprene products were calculated from the ^1^H and ^13^C NMR spectra according to the literature [45].

## 3. Results and Discussion

### 3.1. Synthesis and Structural Characterization of BOXMI-Ligated Rare-Earth Metal Dialkyl Complexes *1*–*3*

**Scheme 1:**
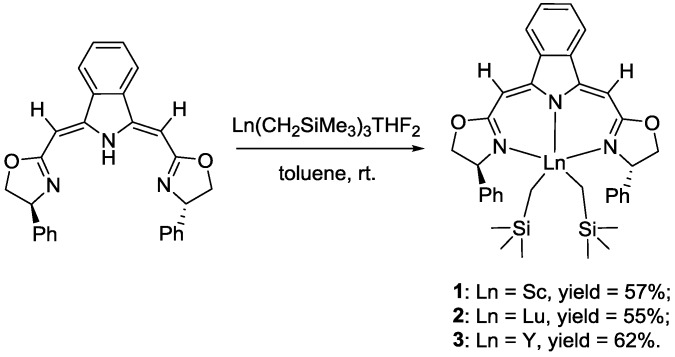
Synthesis of BOXMI-H-ligated rare-earth metal dialkyl complexes 1–3. BOXMI: 1,3-bis(oxazolinymethylidene)isoindoline.

The acid–base reactions of the rare-earth metal trialkyl complexes Ln(CH_2_SiMe_3_)_3_(THF)_2_ and 1 equiv. of 1,3-bis(oxazolinymethylidene)isoindoline ligand (BOXMI-H) straightforwardly yielded the pincer-type NNN-ligated rare-earth metal dialkyl complexes (BOXMI)Ln(CH_2_SiMe_3_)_2_ 1–3 (1: Ln = Sc, yield of 57%; 2: Ln = Lu, yield of 55%; 3: Ln = Y, yield of 62%) with moderate yields (Scheme 1).

These complexes 1–3 have good solubilities in common organic solvents such as hexane, toluene, and THF. In the ^1^H NMR spectra of the complexes 1–3 in C_6_D_6_, all of the proton signals attributed to the NNN ligand except for the proton signal assigned to the N−H group were observed, suggesting the generation of a monoanionic NNN-chelating ligand in these complexes. The molar ratio of the integral areas of the signals for the NNN ligand and trimethylsilylmethyl ligand was 1:2 in each case. No THF molecule was detected in either case. Similarly to the rigid (S,S)-bis(oxazolinylphenyl)amine-ligated rare-earth metal dialkyl complexes, all of the methylene protons of the Ln–CH_2_SiMe_3_ groups showed two doublets at a high field for 1 of 0.16 (d, 2H) and −0.56 ppm (d, 2H), for 2 at −0.62 (d, 2H) and −1.44 ppm (d, 2H), and for 3 at −0.41 (d, 2H) and −1.30 ppm (d, 2H) with a germinal H–H coupling constant of 12 Hz, respectively. These results may suggest that these complexes also have a rigid structure and the CH_2_SiMe_3_ groups in these complexes are fixed to some extent at the NMR time scale.

### 3.2. Single Crystals of Complexes *1* and *2*

**Figure 1:**
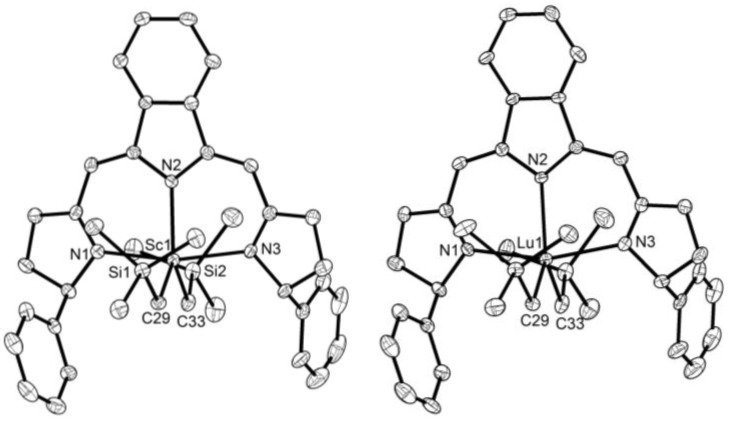
ORTEP drawings of complexes 1 and 2 with thermal ellipsoids of a 30% probability. Hydrogen atoms are omitted for clarity. ORTEP: Oak Ridge Thermal Ellipsoid Plot.

In the glovebox, single crystals of the complexes 1 and 2 suitable for an X-ray determination were grown from a mixed hexane/toluene solution at −30 °C. The ORTEP (Oak Ridge Thermal Ellipsoid Plot) drawings of the complexes 1–2 are shown in Figure 1. The selected bond distances and angles of these complexes 1 and 2 are summarized in Table 1. The X-ray diffraction study revealed that the dialkyl complexes 1 and 2 are isomorphous and isostructural. Both of these complexes contain one *C*_2_-symmetric monoanionic tridentate NNN ligand and two trimethylsilylmethyl groups, adopting a distorted trigonal bipyramidal geometry. The bond distances of the chelating Ln−N(1), Ln−N(2), Ln−N(3), and Ln−C(29) as well as the Ln−C(33) bond increase in the order of 1 < 2 because of the ionic radius of the metal center in a trend of Sc (0.89 Å) < Lu (1.00 Å). The Ln−N(2) bonds of complexes 1 and 2 divide the angles of N(1)−Ln−N(3) (161.8(1)°–166.9(1)°) into two almost equal parts, N(1)−Ln−N(2) and N(2)−Ln−N(3) (81.1(1)°–83.9(1)°), implying the N(1), N(2), N(3), and Ln atoms are almost planar.

### 3.3. Cis-1,4-Polymerization of Isoprene by the Complexes *1–3*/Activator/AlR_3_ Catalytic Systems

The neutral complexes 1–3 alone and the complexes 1–3/AlR_3_ binary systems were inactive for the IP polymerization, while the complexes 1–3/activator (such as [Ph_3_C][B(C_6_F_5_)_4_] (A), [PhMe_2_NH][B(C_6_F_5_)_4_] (B) and B(C_6_F_5_)_3_ (C)) binary systems showed very low activities in the polymerization of IP. In the presence of both an activator and AlR_3_, the complexes 1–3 could promote the *cis*-1,4-polymerization of IP similarly to the 1,3-bis(2-pyridylimino)isoindoline-ligated rare-earth metal dialkyl complexes [39], affording the *cis*-1,4-polyisoprenes (CPIPs) with heavy molecular weights (*M*_n_ of up to 710,000 g/mol) and moderate molecular weight distributions (*M*_w_/*M*_n_ = 2.0–4.5). Some representative results are summarized in Table 2. As an activator, the trityl borate A and the anilinium borate B generally exhibited similar activities and *cis*-1,4 selectivities in the IP polymerization, while the neutral borane C was inert under the same conditions (Table 2, entries 1–5 and 7–10). For the Sc complex 1, the *cis*-1,4-PIPs obtained by borate A had heavier molecular weights and narrower molecular weight distributions (Table 2, entries 1–2 and 4–5), while for the Lu and Y complexes 2 and 3, the *cis*-1,4-PIPs obtained by borate A also had a heavier molecular weight but a broader molecular weight distribution (Table 2, entries 7–10). Similarly to the complex 1/activator/Al*^i^*Bu_3_ systems, the complex 1/activator/AlEt_3_ and the complex 1/activator/AlMe_3_ systems also showed moderate activities of around 3 × 10^3^ (g of polymer)/(mol_Ln_ h) and *cis*-1,4 selectivities of around 88% in the IP polymerization, affording the *cis*-1,4-PIPs with lower molecular weights and broader molecular weight distributions (Table 2, entries 1–6). When the IP polymerization catalyzed by the complex 3/A/Al*^i^*Bu_3_ system was carried out at −20 °C, a PIP with higher *cis*-1,4 selectivity (>95%), a heavier molecular weight (*M*_n_ = 350,000 g/mol), and a narrower molecular weight distribution (*M*_w_/*M*_n_ = 1.83) could be obtained, as shown by the ^1^H and ^13^C NMR analysis (Table 2, entry 12). It is noteworthy that the complex 3 exhibited high activities of up to 6.8 × 10^4^ (g of polymer)/(mol_Ln_ h) when the temperature increased to 70 °C (Table 2, entry 14). Only 0.5 h was needed to completely convert 500 equiv of monomer, producing a moderate molecular weight for *C*PIP (*cis*-1,4 selectivity of 85%, *M*_n_ = 200,000 g/mol) with a moderate molecular weight distribution (*M*_w_/*M*_n_ = 3.31).

**Figure 2:**
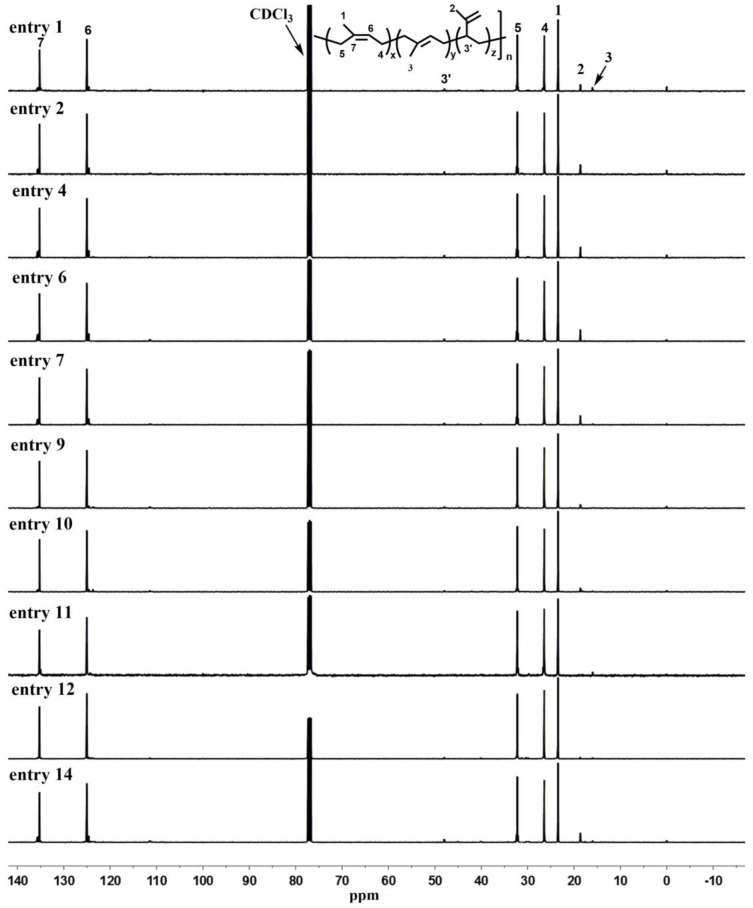
^13^C NMR spectra of the polyisoprenes by complexes 1–3/AlR_3_/borate systems in Table 2.

The resulting PIPs all showed good solubilities in THF and CHCl_3_. The ^1^H NMR spectra of the PIPs obtained by the complex 1–3/activator/AlR_3_ systems in CDCl_3_ indicated the main 1,4-microstructure and a trace amount of a 3,4-microstructure. The ^13^C NMR spectra showed diagnostic signals for a main *cis*-1,4 configuration (δ of 23.4, 26.4, 32.2, 125.0 and 135.2 ppm; *cis*-1,4-PIP selectivity of >90%) and a small amount of 3,4-configuration (δ of 18.6, 26.4, 32.2, 125.0 and 135.2 ppm) with or without a trace amount of *trans*-1,4-configuration (δ of 15.9, 26.4, 32.2, 125.0 and 135.2 ppm; Figure 2). GPC curves revealed that these PIPs had moderate to heavy molecular weights in the range of 77,000–710,000 g/mol and bimodal molecular weight distributions (*M*_w_/*M*_n_ = 2.0–4.5) similar to natural rubber. The DSC curves of the resulting *cis-*1,4-PIPs showed the glass transition temperature in the range of −58 to −65 °C, consistent with the thermoplastic character of the CPIP (see Supplementary Materials).

## 4. Conclusions

In summary, the three pincer-type monoanionic tridentate *C*_2_-symmetric BOXMI-H-ligated rare-earth metal dialkyl complexes 1–3 have been easily synthesized in moderate yields via one-pot acid–base reaction by using the rare-earth metal tris(trimethylsilylmethyl) complexes with the readily available BOXMI-H ligand. The X-ray diffractions demonstrated that the complexes 1 and 2 are isomorphous and isostructural and that each of them adopt a distorted trigonal bipyramidal configuration. Activated by the activators ([Ph_3_C][B(C_6_F_5_)_4_] (**A**), [PhMe_2_NH][B(C_6_F_5_)_4_] (**B**) and B(C_6_F_5_)_3_ (**C**)) and AlR_3_ (R = Me, Et and *^i^*Bu) in toluene, these pincer-type BOXMI-ligated complexes 1–3 exhibited high activities of up to 6.8 × 10^4^ (g of polymer)/(mol_Ln_ h) and high *cis*-1,4 selectivities of up to 97% in the polymerization of isoprene, affording *cis*-1,4-PIPs with heavy molecular weights (*M*_n_ of up to 710,000 g/mol) and bimodal molecular weight distributions (*M*_w_/*M*_n_ = 2.0–4.5). In comparison with the *trans*-1,4-PIPs obtained by the (S,S)-bis(oxazolinylphenyl)amine-ligated rare-earth metal dialkyl complexes [38] and the *cis*-1,4-PIPs obtained by the 1,3-bis(2-pyridylimino)isoindoline-ligated rare-earth metal dialkyl complexes [39], such results demonstrate that the main body skeleton of the chelating ligand has a more important impact on the catalytic performance of these pincer-type rare-earth metal dialkyl complexes in the IP polymerization. Moreover, the rare-earth metal dialkyl complexes bearing the pincer-type chelating ligand with the rigid skeleton and the bulky substituents are not good for the *cis*-1,4-polymerization of IP. These findings will benefit the design of the high-efficiency and selective catalysts, as well as the rapid and precise synthesis of natural rubber. Further studies will be focused on the modification of the chelating ligand to improve the selectivity and/or activity of the rare-earth metal catalytic system in the *cis*-1,4-polymerization of isoprene.

## Figures and Tables

**Table 1:** Selected bond distances (Å) and angles (deg) of complexes 1 and 2.

Bond Lengths and Bond Angles	1	2
Ln−N1	2.272(4)	2.345(4)
Ln−N2	2.228(3)	2.313(4)
Ln−N3	2.274(4)	2.351(4)
Ln−C29	2.235(4)	2.329(5)
Ln−C33	2.241(4)	2.333(5)
N1−Ln−N3	166.9(1)	161.8(1)
N2−Ln−N1	83.8(1)	81.1(1)
N2−Ln−N3	83.9(1)	81.4(1)
N2−Ln−C29	118.9(1)	118.9(1)
N2−Ln−C33	126.3(2)	126.3(4)
C29−Ln−N1	98.3(2)	100.5(2)
C29−Ln−N3	91.6(2)	92.2(3)
C29−Ln−C33	114.8(2)	114.7(2)
C33−Ln−N1	88.3(1)	89.1(2)
C33−Ln−N3	95.4(1)	97.4(8)

**Table 2:**
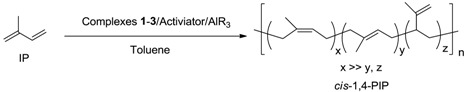
*cis*-1,4-Polymerization of isoprene by complexes 1–3/activator/AlR_3_ ternary systems. *^a^*

Entry	Comp.	Borate *^b^*	AlR_3_	t (h)	T (°C)	Y (%)	A *^c^*	Microstructure (%) *^d^*			*M*_n_ *^e^* (10^4^)	*M*_w_/*M*_n_ *^e^*	*T*_g_ *^f^* (°C)
								*c*-1,4-	*t*-1,4-	3,4-			
1	**1**	A	Al*^i^*Bu_3_	0.5	25	85	23	87	2	11	71	2.00	−58
2	**1**	B	Al*^i^*Bu_3_	4	25	96	3	87	0	13	32	2.75	−60
3	**1**	C	Al*^i^*Bu_3_	12	25	—	—	—	—	—	—	—	—
4	**1**	A	AlEt_3_	4	25	93	3	88	0	12	16	3.18	−59
5	**1**	B	AlEt_3_	4	25	86	3	88	0	12	13	3.21	−62
6	**1**	A	AlMe_3_	6	25	92	2	89	1	10	8	4.31	−58
7	**2**	A	Al*^i^*Bu_3_	4	25	93	3	87	0	13	11	3.72	−59
8	**2**	B	Al*^i^*Bu_3_	4	25	85	3	88	0	12	8	4.51	—
9	**3**	A	Al*^i^*Bu_3_	2	25	92	6	93	0	7	16	1.87	−63
10	**3**	B	Al*^i^*Bu_3_	4	25	85	3	90	0	10	10	3.36	−61
11	**3**	A	Al*^i^*Bu_3_	2	0	82	6	95	0	5	25	2.32	−62
12	**3**	A	Al*^i^*Bu_3_	2	−20	80	5	97	1	2	35	1.83	−65
13	**3**	A	Al*^i^*Bu_3_	0.5	50	93	25	87	0	13	16	2.87	−61
14	**3**	A	Al*^i^*Bu_3_	0.5	70	99	68	85	2	13	20	3.31	−59

*^a^* Conditions unless specified otherwise: 20 μmol of Ln complex, 20 μmol of activator, 10 equiv. of AlR_3_, 4 mmol of isoprene, and 8 mL of toluene. *^b^* Activator: **A** = [Ph_3_C][B(C_6_F_5_)_4_]; **B** = [PhNHMe_2_][B(C_6_F_5_)_4_]; **C** = B(C_6_F_5_)_3_. *^c^* Activity in (kg of polymer)/(mol_Ln_ h). *^d^* Determined by ^1^H and ^13^C NMR spectrum: *c*-1,4, *cis*-1.4 selectivity; *t*-1,4, *trans*-1.4 selectivity; 3,4, 3,4 selectivity. *^e^* Determined by gel permeation chromatography (GPC) in THF at 40 °C against polystyrene standard. *^f^* Measured by differential scanning calorimetry (DSC). *^g^* 10 mmol of isoprene.

## Supplementary Materials

The following are available online at www.mdpi.com/2073-4360/9/10/531/s1, Figure S1: ^1^H NMR spectrum of ligand 6; Figure S2: ^1^H NMR spectrum of ligand 5; Figure S3: ^1^H NMR spectrum of ligand 4; Figure S4: ^1^H NMR spectrum of the complex of 1; Figure S5: ^13^C NMR spectrum of the complex of 1; Figure S6: ^1^H NMR spectrum of the complex of 2; Figure S7: ^13^C NMR spectrum of the complex of 2; Figure S8: ^1^H NMR spectrum of the complex of 3; Figure S9: ^13^C NMR spectrum of the complex of 3; Figure S10. ^1^H NMR spectra of the PIPs by complexes 1-3/AlR_3_/Borate systems in Table 2; Figure S11: ^13^C NMR spectra of the PIPs by complexes 1-3/AlR_3_/Borate systems in Table 2; Figure S12: GPC profiles of the PIPs by complexes 1/Al*^i^*Bu_3_/[Ph_3_C][B(C_6_F_5_)_4_] systems in Table 2, entry1; Figure S13: GPC profiles of the PIPs by complexes 1/Al*^i^*Bu_3_/[PhMe_2_NH][B(C_6_F_5_)_4_] systems in Table 2, entry 2; Figure S14: GPC profiles of the PIPs by complexes 1/AlEt_3_/[Ph_3_C][B(C_6_F_5_)_4_] systems in Table 2, entry 4; Figure S15: GPC profiles of the PIPs by complexes 1/AlEt_3_/[PhMe_2_NH][B(C_6_F_5_)_4_] systems in Table 2, entry 5; Figure S16: GPC profiles of the PIPs by complexes 1/AlMe_3_/[Ph_3_C][B(C_6_F_5_)_4_] systems in Table 2, entry 6; Figure S17: GPC profiles of the PIPs by complexes 2/Al*^i^*Bu_3_/[Ph_3_C][B(C_6_F_5_)_4_] systems in Table 2, entry 7; Figure S18: GPC profiles of the PIPs by complexes 2/Al*^i^*Bu_3_/[PhMe_2_NH][B(C_6_F_5_)_4_] systems in Table 2, entry 8; Figure S19: GPC profiles of the PIPs by complexes 3/Al*^i^*Bu_3_/[Ph_3_C][B(C_6_F_5_)_4_] systems in Table 2, entry 9; Figure S20. GPC profiles of the PIPs by complexes 3/Al*^i^*Bu_3_/[PhMe_2_NH][B(C_6_F_5_)_4_] systems in Table 2, entry 10; Figure S21: GPC profiles of the PIPs by complexes 3/Al*^i^*Bu_3_/[Ph_3_C][B(C_6_F_5_)_4_] systems at 0 °C in Table 2, entry11; Figure S22: GPC profiles of the PIPs by complexes 3/Al*^i^*Bu_3_/[Ph_3_C] [B(C_6_F_5_)_4_] systems at –20 °C in Table 2, entry 12 Figure S23: GPC profiles of the PIPs by complexes 3/Al*^i^*Bu_3_/[Ph_3_C][B(C_6_F_5_)_4_] systems at 50 °C in Table 2, entry13; Figure S24: GPC profiles of the PIPs by complexes 3/Al*^i^*Bu_3_/[Ph_3_C][B(C_6_F_5_)_4_] systems at 70 °C in Table 2, entry 14; Figure S25: DSC charts of the PIPs by complexes 1/Al*^i^*Bu_3_/[Ph_3_C][B(C_6_F_5_)_4_] systems in Table 2, entry 1; Figure S26: DSC charts of the PIPs by complexes 2/Al*^i^*Bu_3_/[PhMe_2_NH][B(C_6_F_5_)_4_] systems in Table 2, entry 2; Figure S27: DSC charts of the PIPs by the 2/AlEt_3_/[Ph_3_C][B(C_6_F_5_)_4_] systems in Table 2, entry 4; Figure S28: DSC charts of the PIPs by the 2/AlEt_3_/[PhMe_2_NH][B(C_6_F_5_)_4_] systems in Table 2, entry 5; Figure S29: DSC charts of the PIPs by the 3/Al*^i^*Bu_3_/[Ph_3_C][B(C_6_F_5_)_4_] systems at 0 °C in Table 2, entry 11; Figure S30. DSC charts of the PIPs by the 3/Al*^i^*Bu_3_/[PhMe_2_NH][B(C_6_F_5_)_4_] systems at –20 °C in Table 2, entry 12; Table S1: Crystal data, data collection and processing parameters for complexes 1 and 2.
