# Supplementary material for: Cis-1,4-Polymerization of Isoprene by 1,3-Bis(oxazolinymethylidene)isoindoline-Ligated Rare-Earth Metal Dialkyl Complexes
Source: Polymers (Basel). 2017 Oct 20;9(10):531. doi: 10.3390/polym9100531 (PMC6419008; doi:10.3390/polym9100531)
Supplement: Supplementary file 1 [file polymers-09-00531-s001.pdf]

Supplementary Materials: Cis-1,4-Polymerization of Isoprene Catalyzed by 1,3-Bis(oxazolinymethylidene)isoindoline Ligated Rare Earth Metal Complex

Chao Yu, Dahai Zhou, Xiangqian Yan, Fei Gao, Li Zhang, Shaowen Zhang*, Xiaofang Li*

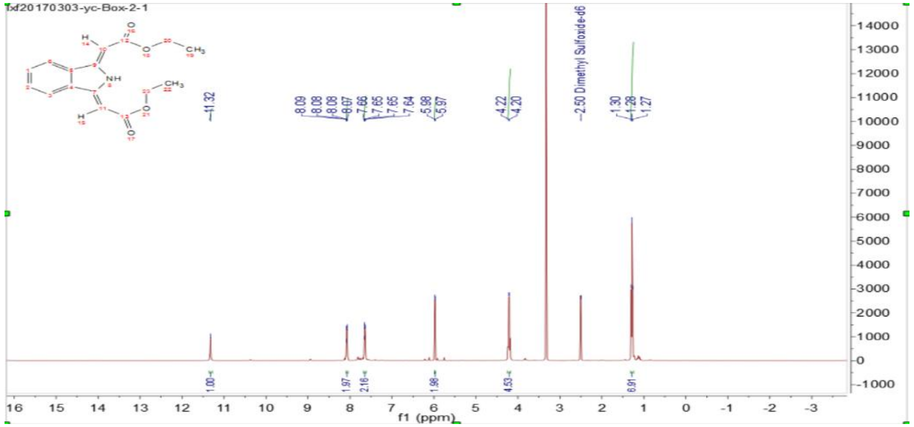

Figure S1. ^{1}H NMR spectrum of ligand **6**

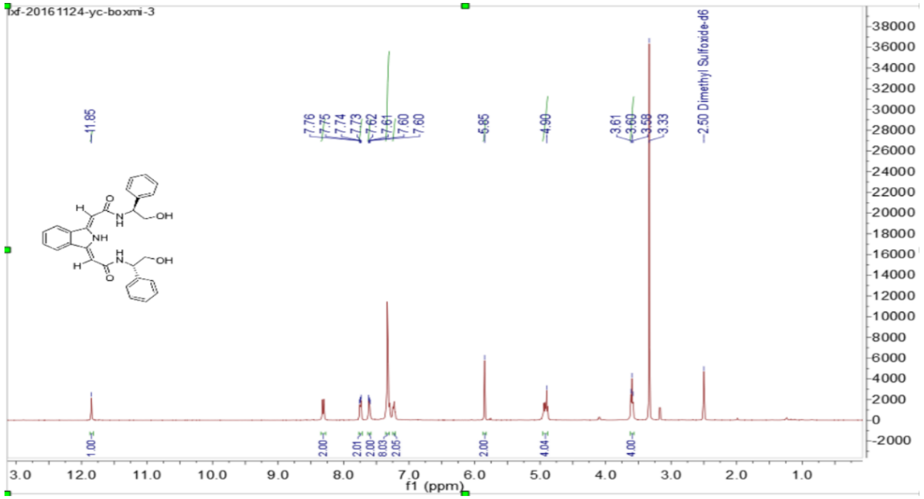

Figure S2. ^{1}H NMR spectrum of ligand **5**

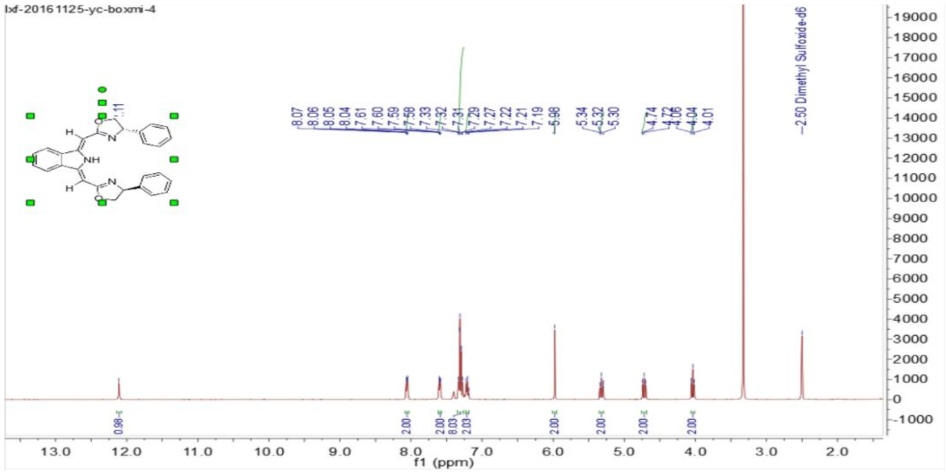

Figure S3. ^{1}H NMR spectrum of ligand **4**

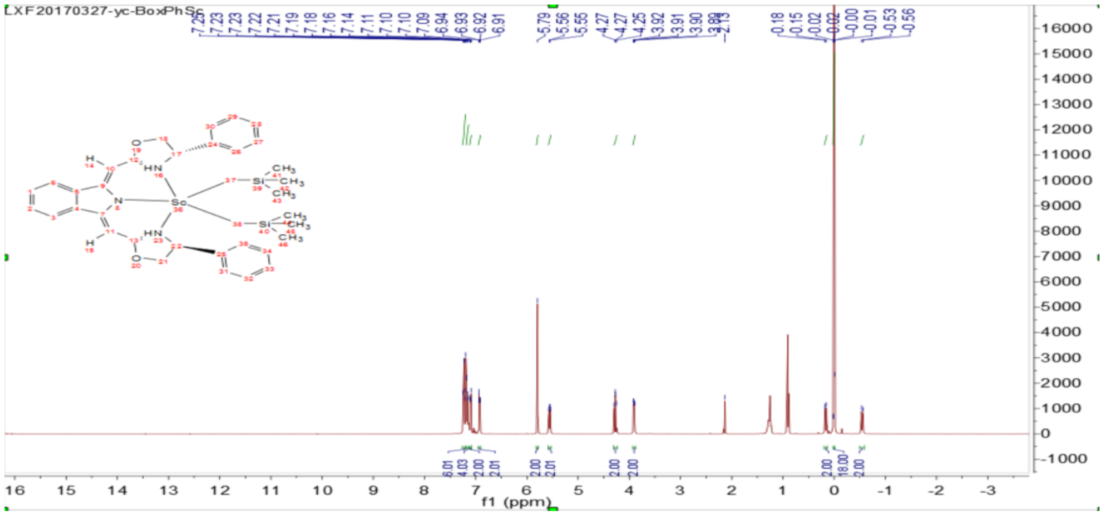

Figure S4. ^{1}H NMR spectrum of complex **1**

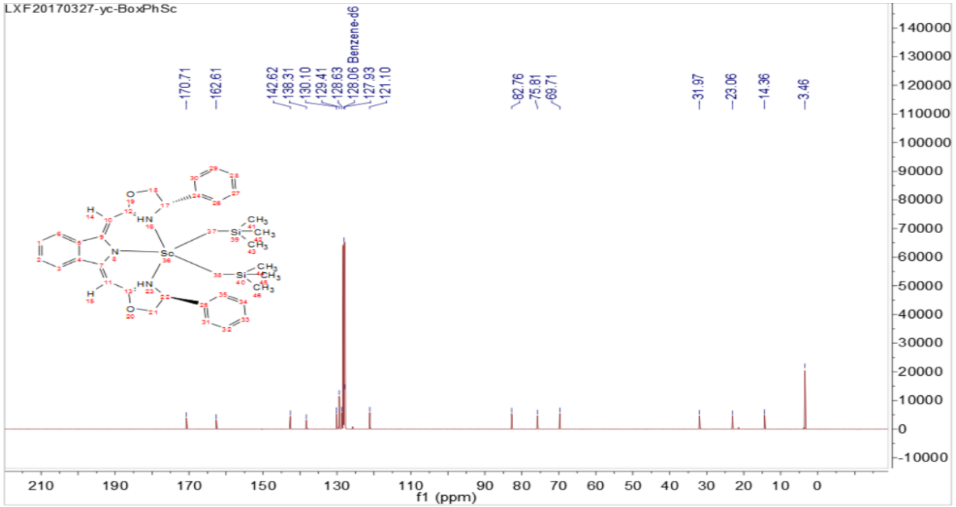

Figure S5. ^{13}C NMR spectrum of complex **1**

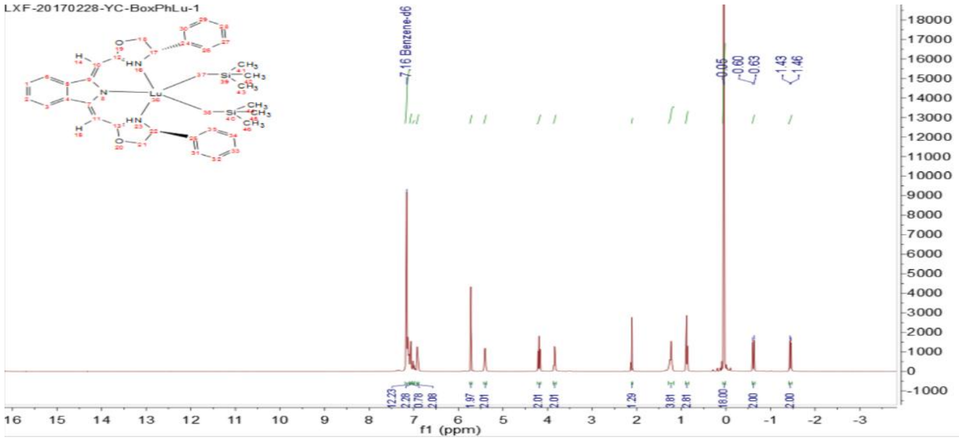

Figure S6. ^{1}H NMR spectrum of complex **2**

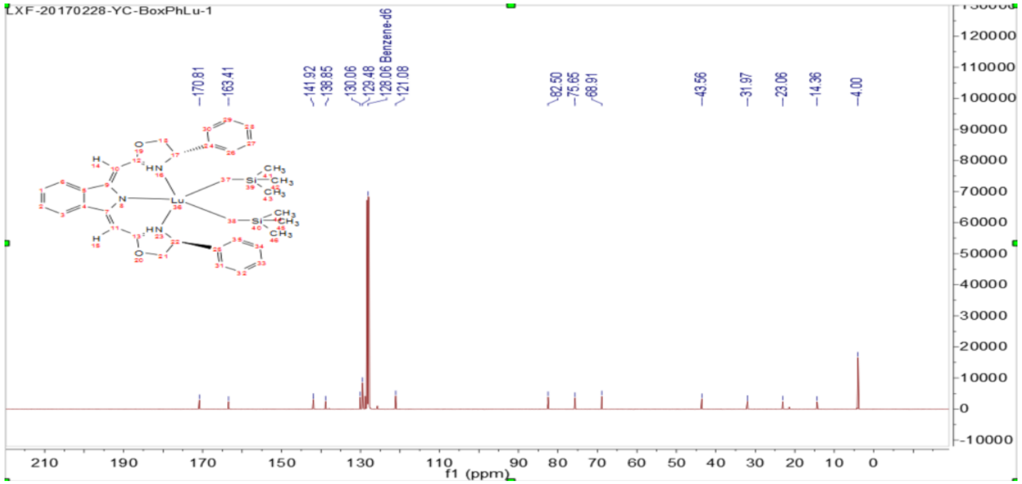

Figure S7. ^{13}C NMR spectrum of complex **2**

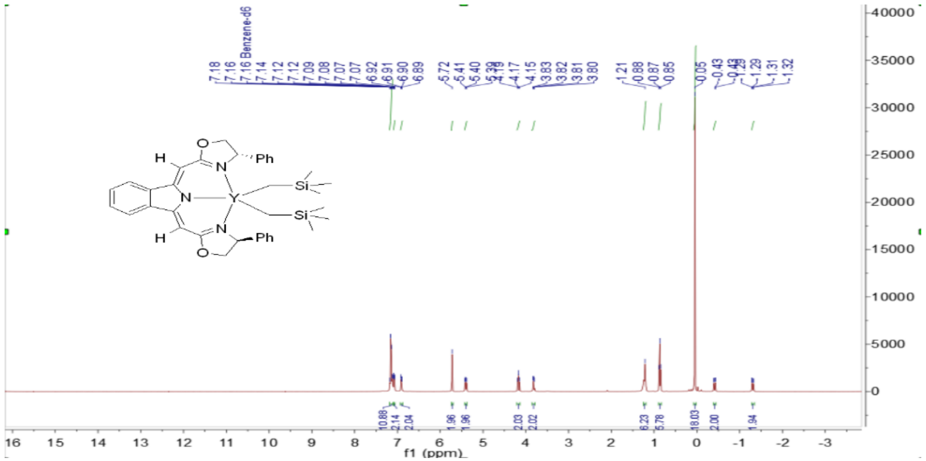

Figure S8. ^{1}H NMR spectrum of complex **3**

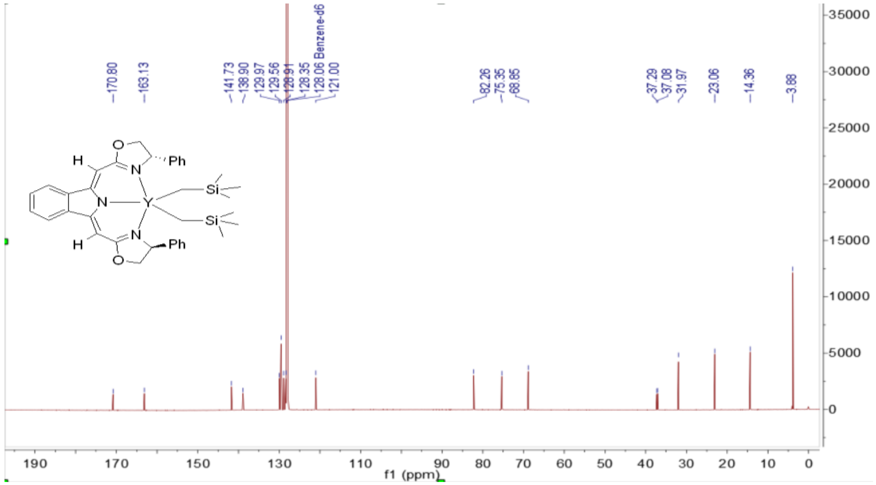

Figure S9. ^{13}C NMR spectrum of complex **3**

A Typical Procedure for the Polymerization of Isoprene by complexes 1/Activator/AlR_3 systems

In a glovebox, to a toluene solution (10 ml) was slowly added to AlR_3 (1M Tol) (0.02 mmol) under magnetic stirring in a flask , then complexes **1-3** (0.02 mmol) was added. After the mixture was stirred for 5 min, a toluene solution (1.0 mL) of Borate (0.02 mmol) was slowly added to the resulting solution, and isoprene (0.39 g, 4 mmol) were added. The polymerization was quenched by addition of ethanol (30 mL, containing 5% butylhydroxytoluene (BHT) as a stabilizing agent). Then the mixture was poured into ethanol (100 mL) to precipitate the polymer product. The precipitated polymer was dried under vacuum at 40 °C to a constant weight.

The isomer contents of the polyisoprene products were calculated from the ^{1}H and ^{13}C NMR spectra according to the following formula (1–5):

(1) Mol 1,4-IP%=$\{I_{H1}/(I_{H1}+0.5I_{H2})\}*100$

(2) Mol 3,4-IP%=$\{0.5I_{H2}/(I_{H1}+0.5I_{H2})\}*100$

in which I_{H1} is the integration of the resonance at 5.13 ppm (one vinyl proton of the 1,4-isoprene unit), and I_{H2} is the integration of the resonance at 4.72 ppm (two vinyl protons of the 3,4-isoprene unit) in the ^{1}H NMR spectrum.

(3) Mol *cis*-1,4-IP%=$\{I_{C1}/(I_{C1}+I_{C2}+I_{C3})\}*100$

(4) Mol *trans*-1,4-IP%=$\{I_{C3}/(I_{C1}+I_{C2}+I_{C3})\}*100$

(5) Mol 3,4-IP%=$\{I_{C2}/(I_{C1}+I_{C2}+I_{C3})\}*100$

in which I_{C1} is the integration of the signals at 23.2 ppm assigned as the methyl carbon of the *cis*-1,4-isoprene unit, and I_{C2} is the integration of the signals at 18.5 ppm assigned as the methyl carbon of the 3,4-isoprene unit, while I_{C3} is the integration of the signals at 15.9 ppm assigned as the methyl carbon of the *trans*-1,4-isoprene unit in the ^{13}C NMR spectrum.

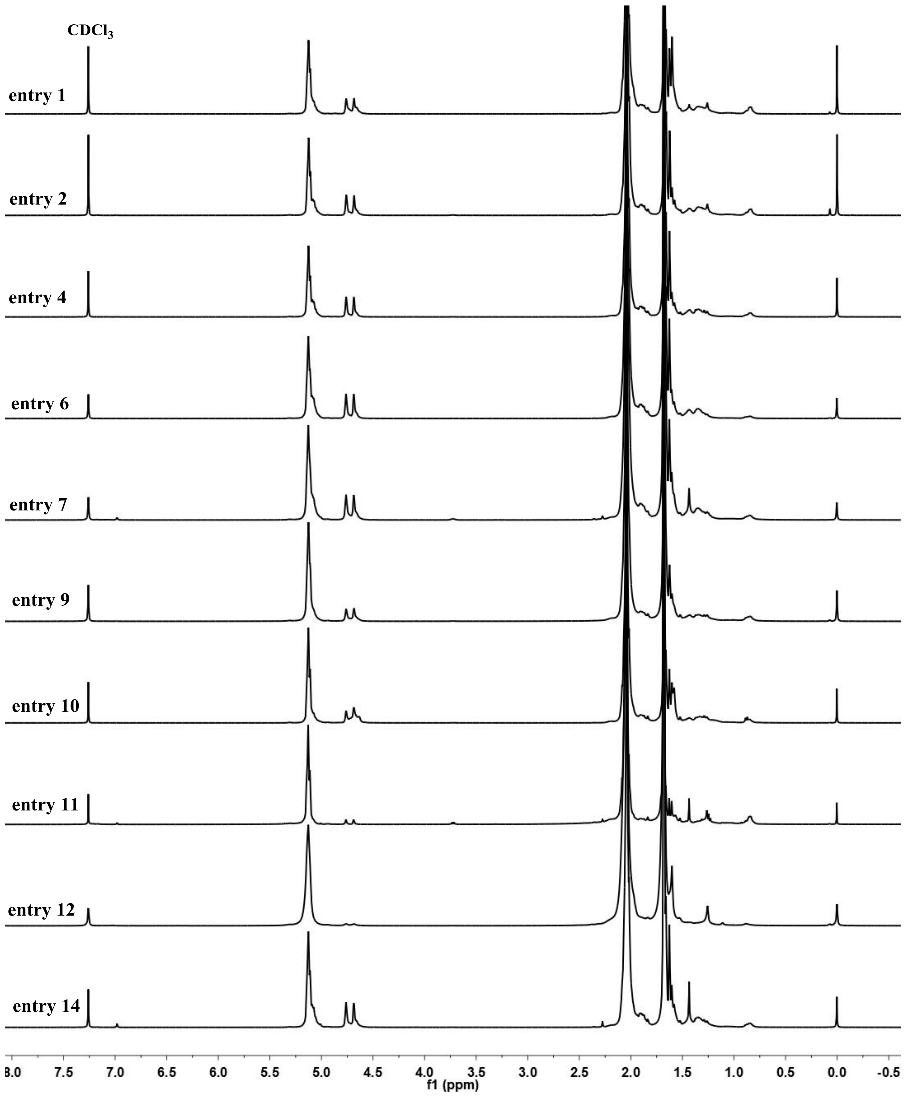

Figure S10. ^{1}H NMR spectra of the polyisoprenes by complexes **1-3**/AlR$_3$/Borate systems in Table 2.

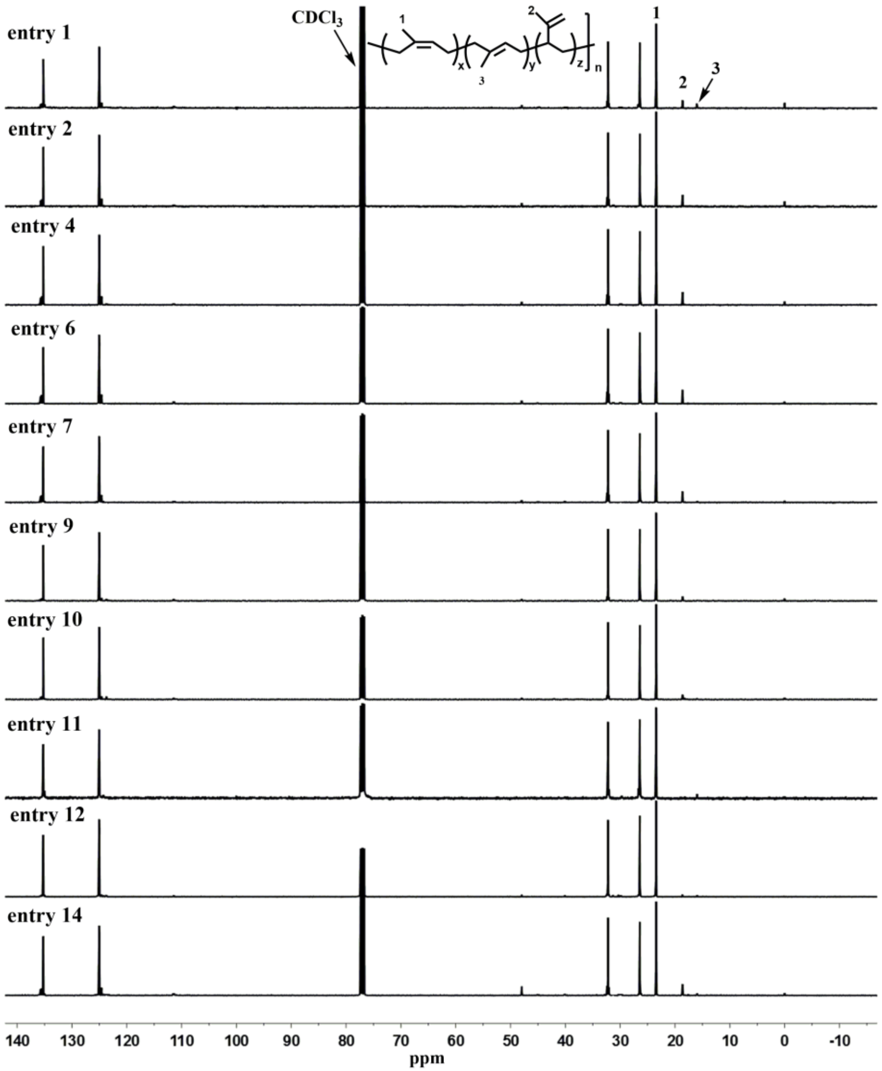

Figure S11. ^{13}C NMR spectra of the polyisoprenes by complexes **1-3**/AlR_3/Borate systems in Table 2.

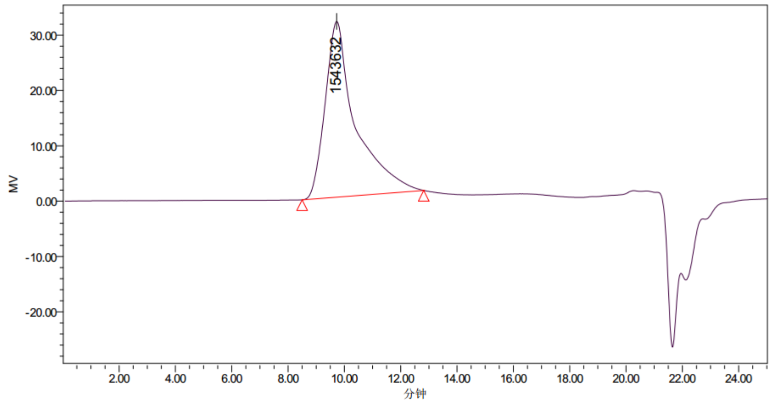

GPC Results

	Dist Name	Elution Volume (ml)	Retention Time (min)	Adjusted RT (min)	Mn	Mw	MP	Mz	Mz+1	Mz/Mw
1		9.728	9.728	9.728	701325	1401672	1543632	2110803	2774290	1.505918

Figure S12. GPC profiles of the polyisoprenes by complexes **1**/Al^{i}Bu$_3$/[Ph$_3$C][B(C$_6$F$_5$)$_4$] systems in Table 2, entry 1.

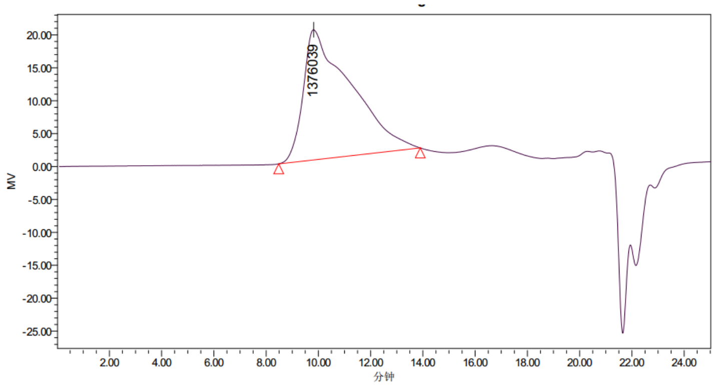

GPC Results

	Dist Name	Elution Volume (ml)	Retention Time (min)	Adjusted RT (min)	Mn	Mw	MP	Mz	Mz+1	Mz/Mw
1		9.816	9.816	9.816	317240	884463	1376039	1724772	2588756	1.950078

Figure S13. GPC profiles of the polyisoprenes by complexes **1**/Al^{i}Bu$_3$/[PhMe$_2$NH][B(C$_6$F$_5$)$_4$] systems in Table 2, entry 2.

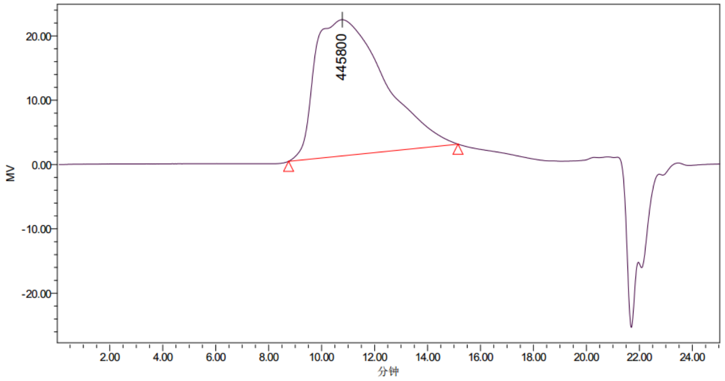

GPC Results

	Dist Name	Elution Volume (ml)	Retention Time (min)	Adjusted RT (min)	Mn	Mw	MP	Mz	Mz+1	Mz/Mw
1		10.778	10.778	10.778	159264	515566	445800	1133170	1783955	2.197914

Figure S14. GPC profiles of the polyisoprenes by complexes **1**/$AlEt_3$/$[Ph_3C][B(C_6F_5)_4]$ systems in Table 2, entry 4.

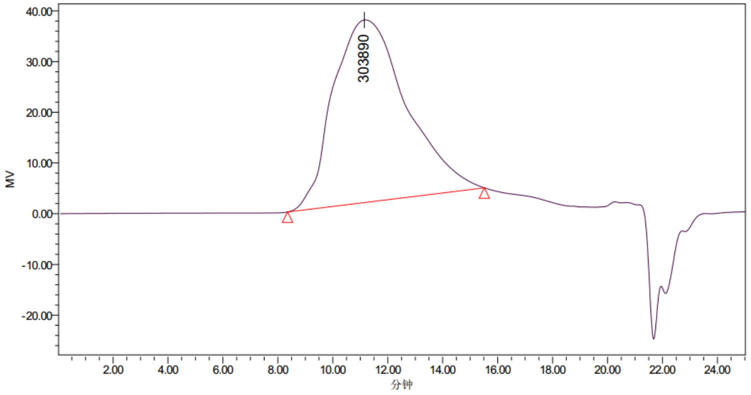

GPC Results

	Dist Name	Elution Volume (ml)	Retention Time (min)	Adjusted RT (min)	Mn	Mw	MP	Mz	Mz+1	Mz/Mw
1		11.150	11.150	11.150	132817	457773	303890	1367561	2899672	2.987425

Figure S15. GPC profiles of the polyisoprenes by complexes **1**/$AlEt_3$/$[PhMe_2NH][B(C_6F_5)_4]$ systems in Table 2, entry 5.

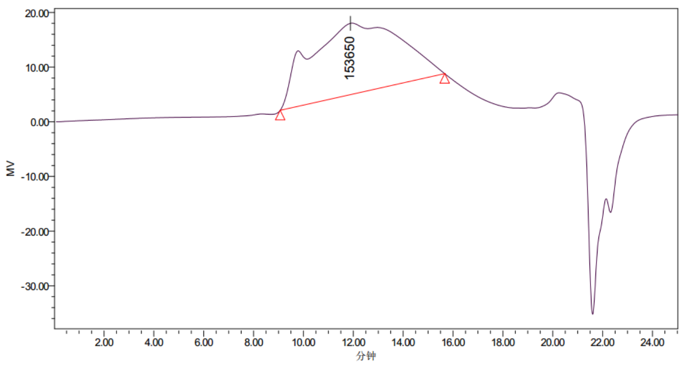

GPC Results

	Dist Name	Elution Volume (ml)	Retention Time (min)	Adjusted RT (min)	Mn	Mw	MP	Mz	Mz+1	Mz/Mw
1		11.883	11.883	11.883	77333	358893	153650	1129264	1709158	3.146522

Figure S16. GPC profiles of the polyisoprenes by complexes **1**/$AlMe_3$/$[Ph_3C][B(C_6F_5)_4]$ systems in Table 2, entry 6.

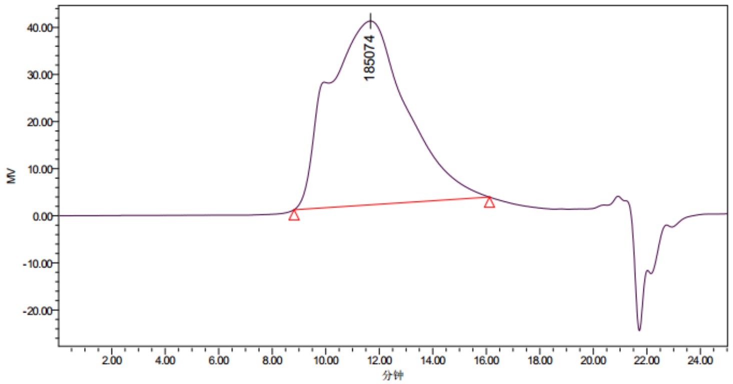

GPC Results

	Dist Name	Elution Volume (ml)	Retention Time (min)	Adjusted RT (min)	Mn	Mw	MP	Mz	Mz+1	Mz/Mw
1		11.673	11.673	11.673	108231	415205	185074	1130915	1842397	2.723752

Figure S17. GPC profiles of the polyisoprenes by complexes **2**/Al^iBu_3/$[Ph_3C][B(C_6F_5)_4]$ systems in Table 2, entry 7.

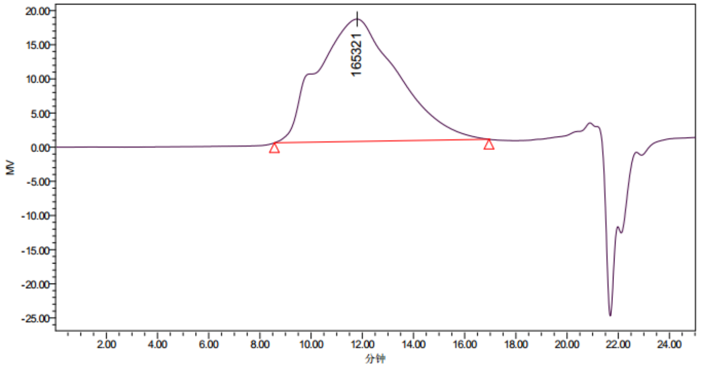

GPC Results

	Dist Name	Elution Volume (ml)	Retention Time (min)	Adjusted RT (min)	Mn	Mw	MP	Mz	Mz+1	Mz/Mw
1		11.800	11.800	11.800	81779	370699	165321	1279622	2441804	3.451914

Figure S18. GPC profiles of the polyisoprenes by complexes **2**/$Al^{i}Bu_3$/$[PhMe_2NH][B(C_6F_5)_4]$ systems in Table 2, entry 8.

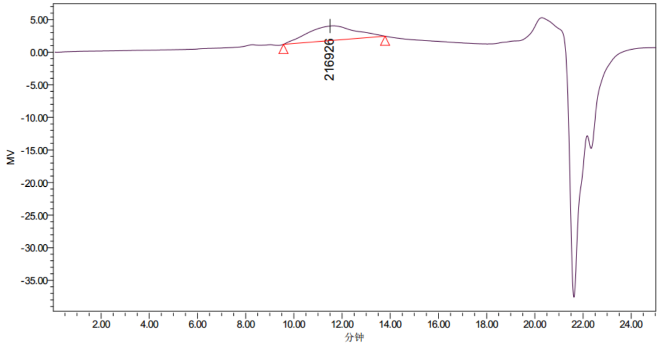

GPC Results

	Dist Name	Elution Volume (ml)	Retention Time (min)	Adjusted RT (min)	Mn	Mw	MP	Mz	Mz+1	Mz/Mw
1		11.500	11.500	11.500	162031	302104	216926	545294	820360	1.804988

Figure S19. GPC profiles of the polyisoprenes by complexes **3**/$Al^{i}Bu_3$/$[Ph_3C][B(C_6F_5)_4]$ systems in Table 2, entry 9.

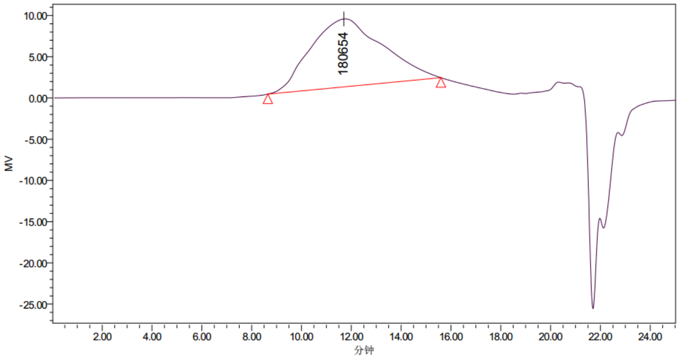

GPC Results

	Dist Name	Elution Volume (ml)	Retention Time (min)	Adjusted RT (min)	Mn	Mw	MP	Mz	Mz+1	Mz/Mw
1		11.700	11.700	11.700	94914	328415	180654	1031563	2079009	3.141034

Figure S20. GPC profiles of the polyisoprenes by complexes **3**/Al^{i}Bu$_3$/[PhMe$_2$NH][B(C$_6$F$_5$)$_4$] systems in Table 2, entry 10.

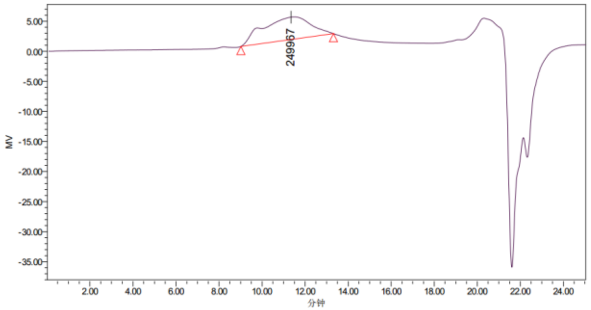

GPC Results

	Dist Name	Elution Volume (ml)	Retention Time (min)	Adjusted RT (min)	Mn	Mw	MP	Mz	Mz+1	Mz/Mw
1		11.350	11.350	11.350	247178	584358	249967	1230223	1807528	2.105258

Figure S21. GPC profiles of the polyisoprenes by complexes **3**/Al^{i}Bu$_3$/[Ph$_3$C][B(C$_6$F$_5$)$_4$] systems at 0 ℃ in Table 2, entry 11.

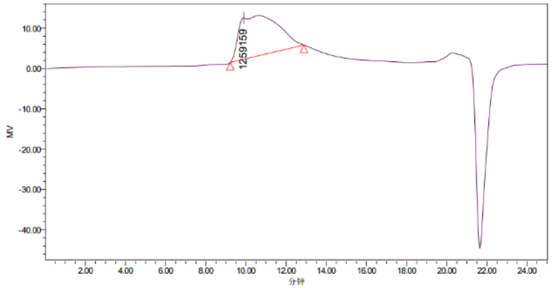

GPC Results

	Dist Name	Elution Volume (ml)	Retention Time (min)	Adjusted RT (min)	Mn	Mw	MP	Mz	Mz+1	Mz/Mw
1		9.886	9.886	9.886	352174	668992	1259159	1081805	1418625	1.617067

Figure S22. GPC profiles of the polyisoprenes by complexes **3**/$Al^{i}Bu_3$/$[Ph_3C][B(C_6F_5)_4]$ systems at –20 ℃ in Table 2, entry 12.

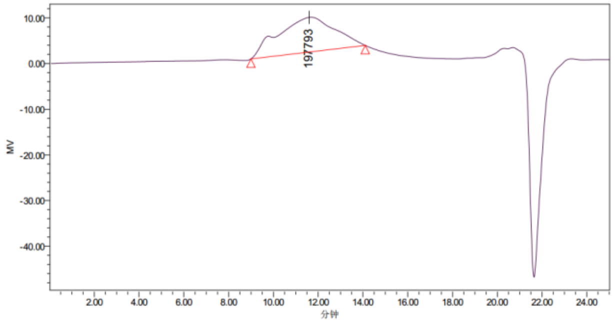

GPC Results

	Dist Name	Elution Volume (ml)	Retention Time (min)	Adjusted RT (min)	Mn	Mw	MP	Mz	Mz+1	Mz/Mw
1		11.600	11.600	11.600	160485	466143	197793	1187687	1849830	2.547902

Figure S23. GPC profiles of the polyisoprenes by complexes **3**/$Al^{i}Bu_3$/$[Ph_3C][B(C_6F_5)_4]$ systems at 50 ℃ in Table 2, entry 13.

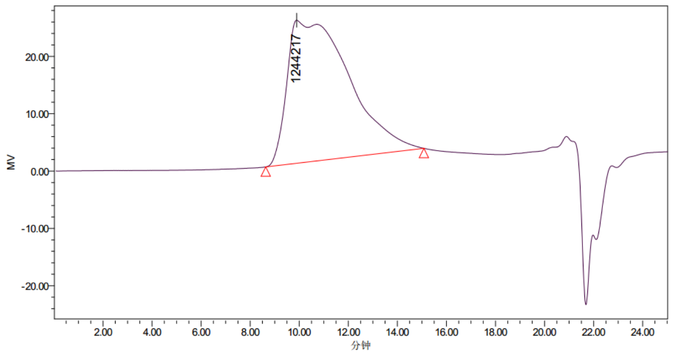

GPC Results

	Dist Name	Elution Volume (ml)	Retention Time (min)	Adjusted RT (min)	Mn	Mw	MP	Mz	Mz+1	Mz/Mw
1		9.896	9.896	9.896	196725	658815	1244217	1434135	2177394	2.176841

Figure S24. GPC profiles of the polyisoprenes by complexes **3**/Al^{i}Bu$_3$/[Ph$_3$C][B(C$_6$F$_5$)$_4$] systems at 70 °C in Table 2, entry 14.

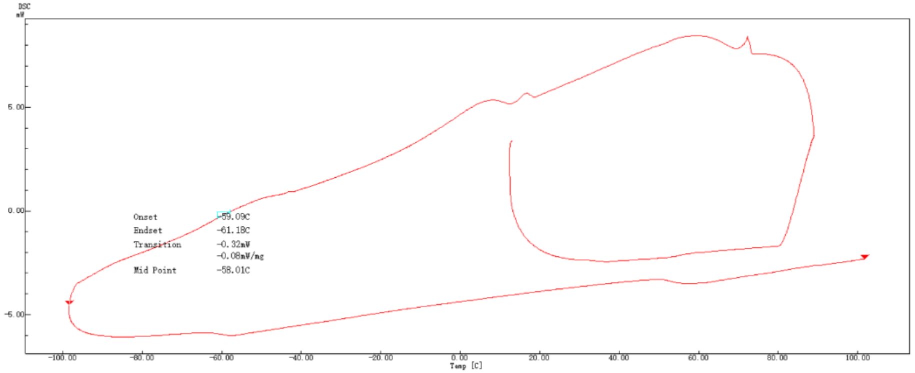

Figure S25. DSC charts of the polyisoprenes by complexes **1**/Al^{i}Bu$_3$/[Ph$_3$C][B(C$_6$F$_5$)$_4$] systems in Table 2, entry 1.

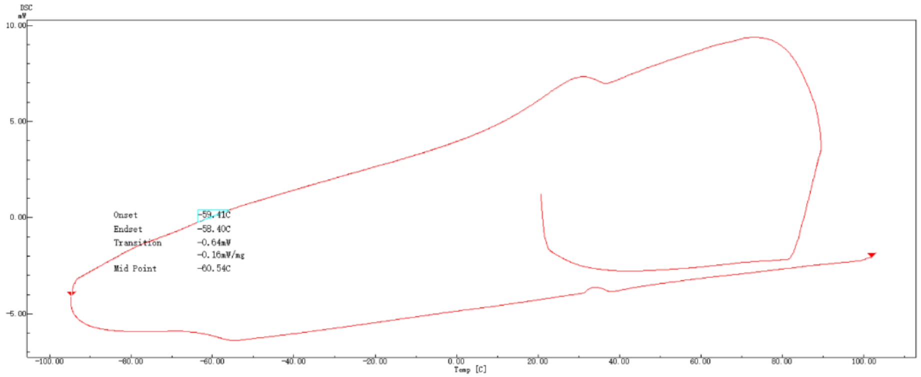

Figure S26. DSC charts of the polyisoprenes by complexes **1**/Al^{i}Bu$_3$/[PhMe$_2$NH][B(C$_6$F$_5$)$_4$] systems in Table 2, entry 2.

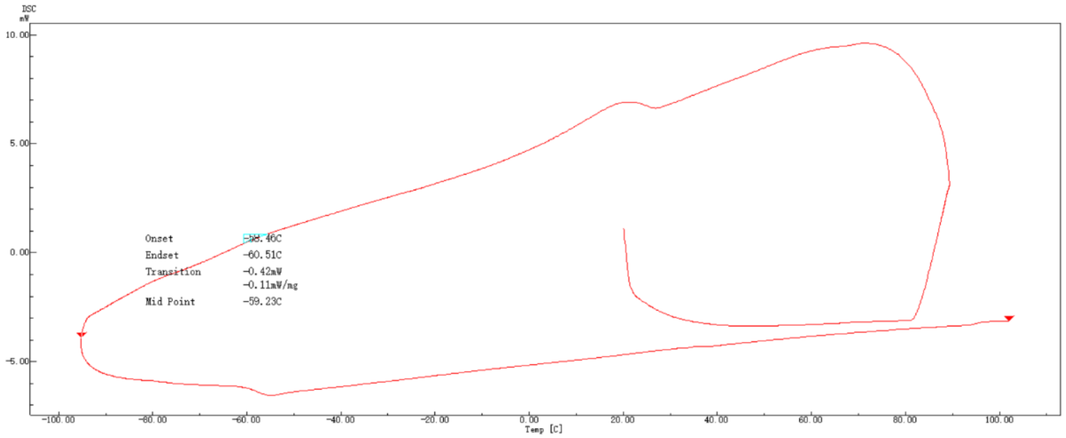

Figure S27. DSC charts of the polyisoprenes by the **1**/AlEt$_3$/[Ph$_3$C][B(C$_6$F$_5$)$_4$] systems in Table 2, entry 4.

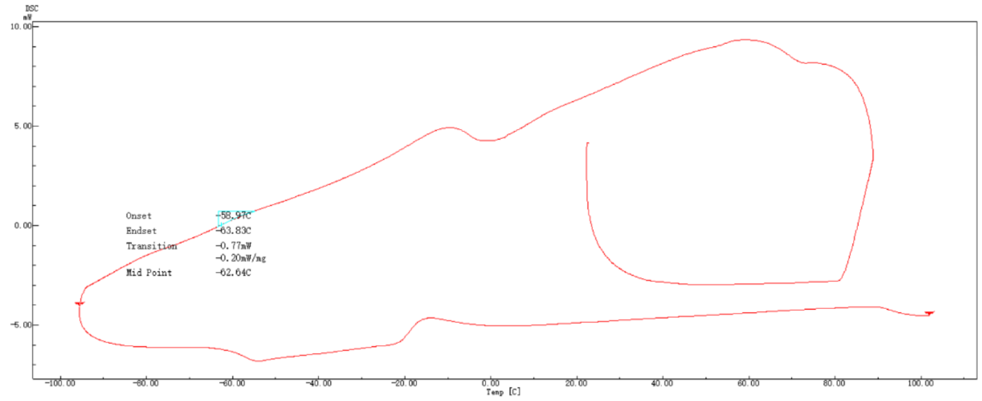

Figure S28. DSC charts of the polyisoprenes by the **1**/AlEt$_3$/[PhMe$_2$NH][B(C$_6$F$_5$)$_4$] systems in Table 2, entry 5.

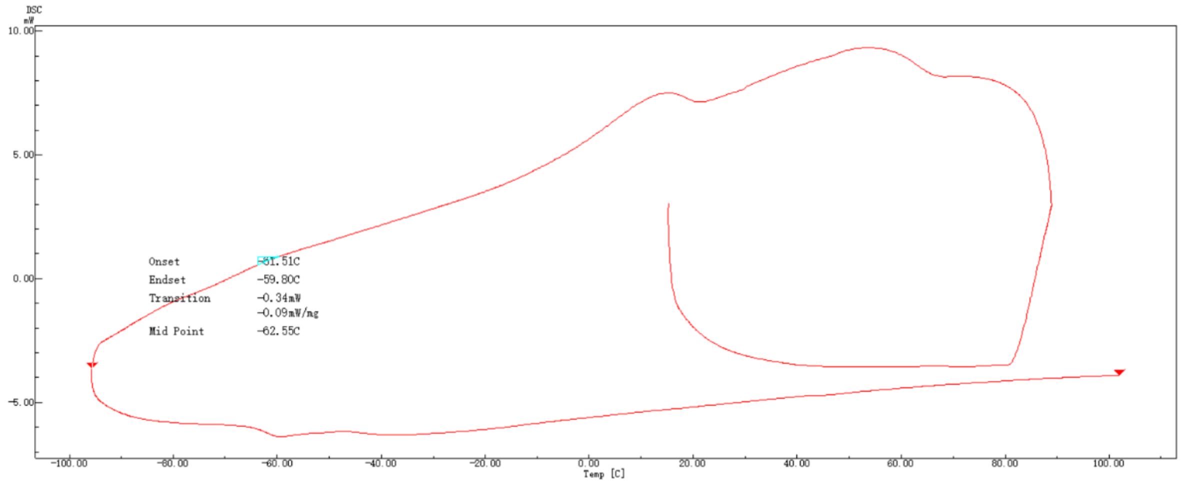

Figure S29. DSC charts of the polyisoprenes by the **3**/Al^{i}Bu$_3$/[PhMe$_2$NH][B(C$_6$F$_5$)$_4$] systems at 0 ℃ in Table 2, entry 11.

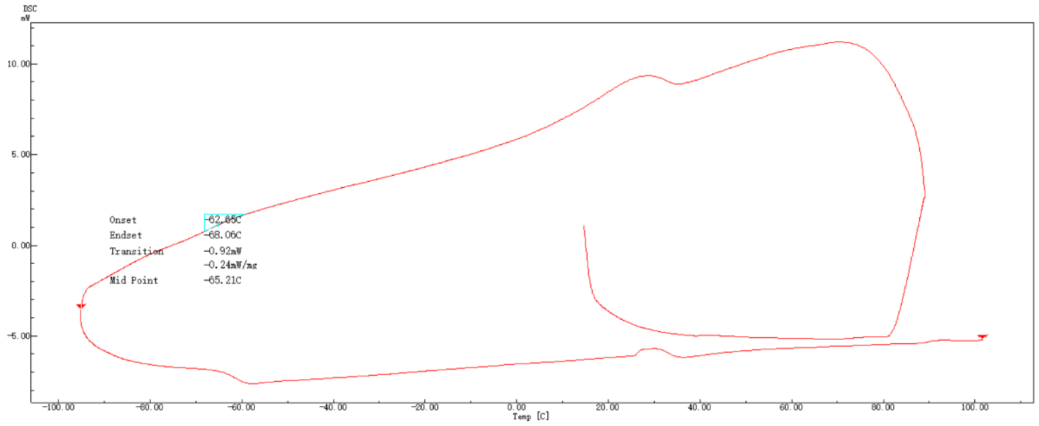

Figure S30. DSC charts of the polyisoprenes by the **3**/Al^{i}Bu$_3$/[Ph$_3$C][B(C$_6$F$_5$)$_4$] systems at −20 °C in Table 2, entry 12.

Table S1. Crystal data, data collection and processing parameters for complexes **1** and **2**.

	**1**	**2**
Formula	$C_{42}H_{58}N_3O_2Si_2Sc$	$C_{36}H_{44}N_3O_2Si_2Lu$
Mw	738.05	781.89
Crystal system	orthorhombic	orthorhombic
Space group	$P2_12_12_1$	$P2_12_12_1$
a [Å]	13.401(3)	13.402(3)
b [Å]	13.601(3)	13.625(3)
c [Å]	23.166(5)	23.318(5)
α [°]	90.00	90.00
β [°]	90.00	90.00
γ [°]	90.00	90.00
V [Å^3]	4222.5(15)	4257.7(15)
Z	4	4
ρ_{calcd} [gcm^{-3}]	1.161	1.220
μ [mm^{-1}]	0.267	2.404
F (000)	1584.0	1584.0
θ range [°]	4.62 to 50	3.46 to 54.98
no of reflns collected	18377	33953

no of indep reflns	7220[R_{int}=0.0425, R_{sigma} = 0.0526]	9670[R_{int}=0.0306, R_{sigma}= 0.0313]
no of date / restraints / params	7220/66/459	9670/54/403
GOF	1.123	1.096
Final R indexes [I>2σ (I)]	R_1=0.0569,wR_2=0.1699	R_1=0.0347,wR_2=0.1038
Final R indexes(all data)	R_1=0.0648,wR_2=0.2008	R_1=0.0356,wR_2=0.1055
Largest diff. peak/hole / e $Å^{-3}$	0.81/-0.54	1.18/-0.83

Crystal Data

Bond length of Complex **1**

Atom	Atom	Length/Å	Atom	Atom	Length/Å
Sc1	N1	2.272(4)	C4	C5	1.396(9)
Sc1	N2	2.228(3)	C5	C6	1.370(7)
Sc1	N3	2.274(4)	C7	C8	1.542(6)
Sc1	C29	2.235(4)	C9	C10	1.441(6)
Sc1	C33	2.241(4)	C10	C11	1.356(6)
Si1	C29	1.839(4	C11	C12	1.483(6)
Si1	C30	1.874(5)	C12	C13	1.377(6)
Si1	C31	1.902(5)	C12	C17	1.396(6)
Si1	C32	1.893(5)	C13	C14	1.395(7)
Si2	C33	1.842(5)	C14	C15	1.393(7)
Si2	C34	1.888(6)	C15	C16	1.389(7)
Si2	C35	1.870(5)	C16	C17	1.382(6)
Si2	C36	1.867(5)	C17	C18	1.466(6)
O1	C20	1.342(5)	C18	C19	1.346(6)
O1	C21	1.453(6)	C19	C20	1.439(6)
O2	C8	1.457(5)	C21	C22	1.522(7)
O2	C9	1.339(5)	C22	C23	1.496(6)
N1	C7	1.490(5)	C23	C24	1.403(7)
N1	C9	1.291(6)	C23	C28	1.382(7)
N3	C20	1.284(5)	C24	C25	1.400(8)

N3	C22	1.503(5)	C25	C26	1.376(11)
N2	C11	1.379(6)	C26	C27	1.353(11)
N2	C18	1.394(5)	C27	C28	1.401(9)
C1	C2	1.387(6)	C37	C38	1.507(14)
C1	C6	1.400(6)	C38	C39	1.439(14)
C1	C7	1.516(6)	C39	C40	1.580(13)
C2	C3	1.406(7)	C40	C41	1.433(14)
C3	C4	1.394(9)	C41	C42	1.483(14)

Angel of Complex **1**

**Atom**	**Atom**	**Atom**	**Angle/°**	**Atom**	**Atom**	**Atom**	**Angle/°**
N1	Sc1	N3	166.94(13)	O2	C8	C7	103.8(3)
N2	Sc1	N1	83.81(13)	O2	C9	C10	115.3(4)
N2	Sc1	N2	83.92(13)	N1	C9	O2	117.2(4)
N2	Sc1	C29	118.91(14)	N1	C9	C10	127.5(4)
N2	Sc1	C33	126.33(15)	C11	C10	C9	126.1(4)
C29	Sc1	N1	98.28(15)	N2	C11	C12	109.8(4)
C29	Sc1	N3	91.56(15)	C10	C11	N3	128.1(4)
C29	Sc1	C33	114.76(17)	C10	C11	C12	122.0(4)
C33	Sc1	N1	88.33(14)	C13	C12	C11	131.9(4)
C33	Sc1	N3	95.36(14)	C13	C12	C17	122.1(4)
C29	Si1	C30	111.1(2)	C17	C12	C11	106.1(4)
C29	Si1	C31	114.3(2)	C12	C13	C14	117.6(4)
C29	Si1	C32	110.1(2)	C15	C14	C13	120.7(4)
C30	Si1	C31	105.1(2)	C16	C15	C14	121.1(4)
C30	Si1	C32	109.3(2)	C17	C16	C15	118.3(5)
C32	Si1	C31	106.6(2)	C12	C17	C18	107.0(4)
C33	Si2	C34	112.6(2)	C16	C17	C12	120.3(4)
C33	Si2	C35	113.0(3)	C16	C17	C18	132.7(4)
C33	Si2	C36	111.6(2)	N2	C18	C17	109.7(4)

C35	Si2	C34	107.4(3)	C19	C18	N2	127.6(4)
C36	Si2	C34	106.1(3)	C19	C18	C17	122.7(4)
C36	Si2	C35	105.7(3)	C18	C19	C20	126.5(4)
C20	O1	C21	106.6(3)	O1	C20	C19	114.8(4)
C9	O2	C8	107.8(3)	N3	C20	O1	117.0(4)
C7	N1	Sc1	123.9(3)	N3	C20	C19	128.2(4)
C9	N1	Sc1	127.2(3)	O1	C21	C22	105.1(3)
C9	N1	C7	107.5(4)	N2	C22	C21	102.3(3)
C20	N3	Sc1	126.2(3)	C23	C22	N3	112.9(4)
C20	N3	C22	107.7(4)	C23	C22	C21	114.2(4)
C22	N3	Sc1	124.9(3)	C24	C23	C22	120.5(4)
C11	N2	Sc1	126.5(3)	C28	C23	C22	120.8(5)
C11	N2	C18	107.4(3)	C28	C23	C24	118.7(5)
C18	N2	Sc1	126.1(3)	C25	C24	C23	119.8(6)
C2	C1	C6	120.1(4)	C26	C25	C24	119.9(7)
C2	C1	C7	119.4(4)	C27	C26	C25	120.7(6)
C6	C1	C7	120.5(4)	C26	C27	C28	120.3(6)
C1	C2	C3	119.8(5)	C23	C28	C27	120.5(6)
C4	C3	C2	119.5(5)	Si1	C29	Sc1	117.2(2)
C3	C4	C5	119.8(5)	Si2	C33	Sc1	118.2(2)
C6	C5	C4	120.7(5)	C39	C38	C37	116.9(10)
C5	C6	C1	120.1(5)	C38	C39	C40	109.7(10)
N1	C7	C1	111.8(3)	C41	C40	C39	114.3(10)
N1	C7	C8	103.8(3)	C40	C41	C42	117.3(11)
C1	C7	C8	113.6(4)				

Bond length of Complex **2**

Atom	Atom	Length/Å	Atom	Atom	Length/Å
Lu1	N1	2.345(4)	C3	C8	1.400(8)
Lu1	N2	2.313(4)	C4	C5	1.402(8)
Lu1	N3	2.351(4)	C5	C6	1.395(11)
Lu1	C29	2.329(5)	C6	C7	1.369(12)
Lu1	C33	2.333(5)	C7	C8	1.372(8)
Si1	C29	1.855(5)	C9	C10	1.415(7)
Si1	C30	1.903(6)	C10	C11	1.361(7)
Si1	C31	1.894(7)	C11	C12	1.474(6)
Si1	C32	1.892(6)	C12	C13	1.392(7)
Si2	C33	1.853(6)	C12	C17	1.401(7)
Si2	C34	1.880(7)	C13	C14	1.407(8)
Si2	C35	1.886(7)	C14	C15	1.354(8)
Si2	C36	1.861(6)	C15	C16	1.374(8)
O1	C2	1.427(7)	C16	C17	1.395(7)
O1	C9	1.362(6)	C17	C18	1.481(6)
O2	C20	1.343(6)	C18	C19	1.354(7)
O2	C21	1.435(7)	C19	C20	1.432(7)
N1	C1	1.498(6)	C21	C22	1.538(8)
N1	C9	1.285(7)	C22	C23	1.506(8)
N2	C11	1.399(6)	C23	C24	1.381(9)
N2	C18	1.396(6)	C23	C28	1.376(9)
N3	C20	1.276(7)	C24	C25	1.392(11)
N3	C22	1.504(6)	C25	C26	1.338(13)
C1	C2	1.549(7)	C26	C27	1.376(12)
C1	C3	1.501(7)	C27	C28	1.363(9)
C3	C4	1.391(7)			

Angel of Complex **2**

Atom	Atom	Atom	Angle/°	Atom	Atom	Atom	Angle/°
N1	Lu1	N3	161.80(14)	C7	C6	C5	120.3(6)
N2	Lu1	N1	81.05(14)	C6	C7	C8	120.5(7)
N2	Lu1	N3	81.40(14)	C7	C8	C3	121.0(6)
N2	Lu1	C29	118.99(16)	O1	C9	C10	114.7(4)
N2	Lu1	C33	126.33(18)	N1	C9	O1	116.7(5)
C29	Lu1	N1	100.47(16)	N1	C9	C10	128.6(5)
C29	Lu1	N3	92.22(16)	C11	C10	C9	126.4(5)
C29	Lu1	C33	114.7(2)	N2	C11	C12	109.5(4)
C33	Lu1	N1	89.10(16)	C10	C11	N2	128.1(4)
C33	Lu1	N3	97.46(17)	C10	C11	C12	122.3(5)
C29	Si1	C30	109.6(3)	C13	C12	C11	132.0(5)
C29	Si1	C31	110.0(3)	C13	C12	C17	120.9(5)
C29	Si1	C32	114.7(3)	C17	C12	C11	107.2(4)
C31	Si1	C30	109.5(3)	C12	C13	C14	117.2(5)
C32	Si1	C30	106.8(3)	C15	C14	C13	121.8(5)
C32	Si1	C31	106.1(3)	C14	C15	C16	121.3(5)
C33	Si2	C34	109.6(3)	C15	C16	C17	119.0(5)
C33	Si2	C35	113.7(3)	C12	C17	C18	106.1(4)
C33	Si2	C36	112.2(3)	C16	C17	C12	119.9(5)
C34	Si2	C35	108.3(4)	C16	C17	C18	134.0(5)
C36	Si2	C34	106.8(3)	N2	C18	C17	109.9(4)
C36	Si2	C35	105.9(3)	C19	C18	N2	128.4(4)
C9	O1	C2	107.5(4)	C19	C18	C17	121.6(5)
C20	O2	C21	107.8(4)	C18	C19	C20	126.7(5)
C1	N1	Lu1	122.4(3)	O2	C20	C19	114.8(5)
C9	N1	Lu1	128.2(3)	N3	C20	O2	116.7(5)
C9	N1	C1	107.9(4)	N3	C20	C19	128.4(5)
C11	N2	Lu1	126.7(3)	O2	C21	C22	104.6(4)
C18	N2	Lu1	126.0(3)	N3	C22	C21	102.2(4)
C18	N2	C11	107.1(4)	N3	C22	C23	113.4(4)
C20	N3	Lu1	127.3(3)	C23	C22	C21	113.8(5)
C20	N3	C22	107.9(4)	C24	C23	C22	120.7(6)

C22	N3	Lu1	123.7(3)	C28	C23	C22	121.0(5)
N1	C1	C2	102.6(4)	C28	C23	C24	118.3(7)
N1	C1	C3	111.5(4)	C23	C24	C25	120.1(8)
C3	C1	C2	115.3(4)	C26	C25	C24	120.4(8)
O1	C2	C1	105.3(4)	C25	C26	C27	119.9(7)
C4	C3	C1	120.4(5)	C28	C27	C26	120.4(8)
C4	C3	C8	118.7(5)	C27	C28	C23	120.8(7)
C8	C3	C1	120.9(5)	Si1	C29	Lu1	115.9(2)
C3	C4	C5	120.1(6)	Si2	C33	Lu1	117.4(3)
C6	C5	C4	119.5(6)				
